# GRETNA: a graph theoretical network analysis toolbox for imaging connectomics

**DOI:** 10.3389/fnhum.2015.00386

**Published:** 2015-06-30

**Authors:** Jinhui Wang, Xindi Wang, Mingrui Xia, Xuhong Liao, Alan Evans, Yong He

**Affiliations:** ^1^State Key Laboratory of Cognitive Neuroscience and Learning and IDG/McGovern Institute for Brain Research, Beijing Normal UniversityBeijing, China; ^2^Center for Cognition and Brain Disorders, Hangzhou Normal UniversityHangzhou, China; ^3^Zhejiang Key Laboratory for Research in Assessment of Cognitive ImpairmentsHangzhou, China; ^4^McConnell Brain Imaging Center, Montreal Neurological Institute, McGill UniversityMontreal, QC, Canada

**Keywords:** network, graph theory, connectome, resting fMRI, small-world, hub

## Abstract

Recent studies have suggested that the brain’s structural and functional networks (i.e., connectomics) can be constructed by various imaging technologies (e.g., EEG/MEG; structural, diffusion and functional MRI) and further characterized by graph theory. Given the huge complexity of network construction, analysis and statistics, toolboxes incorporating these functions are largely lacking. Here, we developed the GRaph thEoreTical Network Analysis (GRETNA) toolbox for imaging connectomics. The GRETNA contains several key features as follows: (i) an open-source, Matlab-based, cross-platform (Windows and UNIX OS) package with a graphical user interface (GUI); (ii) allowing topological analyses of global and local network properties with parallel computing ability, independent of imaging modality and species; (iii) providing flexible manipulations in several key steps during network construction and analysis, which include network node definition, network connectivity processing, network type selection and choice of thresholding procedure; (iv) allowing statistical comparisons of global, nodal and connectional network metrics and assessments of relationship between these network metrics and clinical or behavioral variables of interest; and (v) including functionality in image preprocessing and network construction based on resting-state functional MRI (R-fMRI) data. After applying the GRETNA to a publicly released R-fMRI dataset of 54 healthy young adults, we demonstrated that human brain functional networks exhibit efficient small-world, assortative, hierarchical and modular organizations and possess highly connected hubs and that these findings are robust against different analytical strategies. With these efforts, we anticipate that GRETNA will accelerate imaging connectomics in an easy, quick and flexible manner. GRETNA is freely available on the NITRC website.^1^

## Introduction

The human brain operates as an interconnected network that responds to various inputs from different sensory systems in real time. A substantial body of evidence suggests that the powerful performance arises from a highly optimized wiring layout embedded in our brains by coordinating neural activities among distributed neuronal populations and brain regions (Mesulam, 1990; McIntosh, 1999; Bressler and Menon, 2010). Mapping and characterization of the underlying structural and functional connectivity patterns of the human brain (i.e., connectomics; Sporns et al., 2005; Biswal et al., 2010) in both typical and atypical population is therefore fundamental since they provide invaluable insights into how the collective of the human brain elements is topologically organized to promote cognitive demands (Park and Friston, 2013) and how the topology dynamically reorganizes to respond to various brain disorders (Bullmore and Sporns, 2009; He and Evans, 2010; Rubinov and Bullmore, 2013).

Recent advances in the human connectomics have shown that human brain networks can be non-invasively obtained from a variety of neurophysiological and neuroimaging techniques, such as electroencephalography/magnetoencephalography (EEG/MEG), functional near infrared spectroscopy (fNIRS), structural MRI, diffusion MRI and functional MRI. Based on data from these modalities, the brain networks can be generally categorized into structural networks and functional networks. Structural brain networks can be constructed by calculating interregional morphological correlations (e.g., cortical thickness) based on structural MRI (He et al., 2007; Bassett et al., 2008; Tijms et al., 2012) or by tracing interregional fiber pathways based on diffusion MRI (Hagmann et al., 2007; Iturria-Medina et al., 2007; Gong et al., 2009). Functional brain networks can be derived by estimating interregional statistical dependences in the BOLD signal from functional MRI (Biswal et al., 1995; Salvador et al., 2005), regional cerebral blood flow from arterial spin labeling (Liang et al., 2014), oxygenated/deoxygenated hemoglobin concentrations from functional near-infrared spectroscopy (fNIRS; Niu et al., 2012) or electrophysiological signals from EEG/MEG (Stam, 2004; Stam et al., 2007). Once the brain networks are constructed, a common mathematical framework based on graph theory can be employed to topologically characterize the organizational principles that govern the networks. In graph theory, a network is abstracted as a graph composed of a collective of nodes linked by edges. For human brain networks, nodes typically represent structurally, functionally or randomly defined regions of interest (ROIs), and edges represent inter-nodal structural or functional connectivity that can be derived from the above-mentioned data modalities.

Recent years have witnessed a surge of interest in the study of human brain networks (Bullmore and Sporns, 2009; Xia and He, 2011; Filippi et al., 2013). In response, several freely available toolboxes have been developed to implement and visualize graph-based topological analyses of brain networks, such as the Brain Connectivity Toolbox (BCT; Rubinov and Sporns, 2010), eConnectome (He et al., 2011), CONN (Whitfield-Gabrieli and Nieto-Castanon, 2012), Graph-Analysis Toolbox (GAT; Hosseini et al., 2012) and GraphVar (Kruschwitz et al., 2015). Specifically, we have previously developed PANDA (Cui et al., 2013) for the construction of structural brain networks based on diffusion imaging data and BrainNet Viewer (Xia et al., 2013) toolkits for the visualization of brain networks. These toolboxes, with distinct advantages and unique scopes of application (Table 1), together tremendously accelerate the progress of brain connectome studies. However, these toolboxes either cover only single functions of network construction, analysis or statistics or are powerless or inflexible in the face of huge computational loads and complex and diverse processes (Table 1, we will return this issue in the “Discussion” Section). A complete, efficient and flexible pipeline toolbox for imaging connectomics is currently lacking.

**Table 1:** **Summary of neuroscience connectomics tools**.

Software	R-fMRI Pre-processing	Network construction (static)	Network construction (dynamic)	Graph analysis	Statistics	Fle	GUI	Parallel computing	Vis	Website
GRETNA	✓	✓	✓	✓	✓	✓	✓	✓	✗	http//www.nitrc.org/projects/gretna/
BCT	✗	✗	✗	✓	✗	✗	✗	✗	✗	https://sites.google.com/site/bctnet/
GAT	✗	✓	✗	✓	✓	✗	✓	✗	✓	Not available
PANDA	✗	✓	✗	✗	✗	✗	✓	✓	✗	http//www.nitrc.org/projects/panda/
CONN	✓	✓	✗	✓	✓	✗	✓	✗	✓	http//www.nitrc.org/projects/conn
eConnectome	✗	✓	✗	✗	✗	✗	✓	✗	✓	http://econnectome.umn.edu/
BrainNet Viewer	✗	✗	✗	✗	✗	✗	✓	✗	✓	http://www.nitrc.org/projects/bnv/
GraphVar	✗	✓	✓	✓	✓	✓	✓	✗	✓	http://www.nitrc.org/projects/graphvar/
Brainwaver	✗	✓	✗	✓	✗	✗	✗	✗	✓	http://cran.r-project.org/web/packages/brainwaver/

*Fle, flexibility; GUI, graphical user interface; Vis, visualization. Note: flexibility is determined according to whether a toolbox provides options regarding at least three of the following factors: network node, network connectivity, network connectivity member, network type and thresholding procedure*.

Here, we developed the GRaph thEoreTical Network Analysis (GRETNA) toolbox to perform comprehensive graph-based topological analyses of brain networks. The GRETNA is a Matlab-based, open-source package with a graphical user interface (GUI). Compared with previous toolboxes, the most impressive features of GRETNA are the combination of multiple functional modules, flexible manipulation and parallel computation (Table 1). Specifically, GRETNA incorporates network construction, analysis and comparison modules to provide a complete and automatic pipeline for connectomics. Given the popularity of resting-state functional MRI (R-fMRI) in mapping intrinsic brain connectivity patterns and studying the topological architecture of diseased brains (Biswal et al., 1995; Fox and Raichle, 2007; Van Dijk et al., 2010; Wang et al., 2010), GRETNA exclusively extends the capabilities for R-fMRI data preprocessing and subsequent network construction procedures. Moreover, GRETNA enables an easy, quick and flexible manner to manipulate different network analytical strategies, including structurally, functionally or randomly defined network nodes, positive or negative connectivity processing, binary or weighted network types and the choices of different thresholding procedures or ranges. Finally, GRETNA is capable of performing parallel computing in the network construction and analysis modules, an intriguing feature that can substantially shorten the duration of network analyses of large data sets. With these efforts, we anticipate that this toolbox will facilitate graph-based brain network studies, particularly those based on R-fMRI data. Currently, the Gretna has been successfully applied to many previous connectome studies (He et al., 2008; Wang et al., 2011, 2015; Cao et al., 2013; Zhong et al., 2015).

## Materials and Methods

### Overview of Functionality of GRETNA

GRETNA is an open-source, Matlab-based, cross-platform (Windows and UNIX OS) package under General Public License (GPL) that provides a GUI framework to implement comprehensive graph-based analyses of network topology, perform statistical comparisons of between-group differences in network metrics and examine the relationships between network properties and other variables of interest. It is worth emphasizing that these functionalities are applicable to any connectivity networks that are derived from various toolboxes (e.g., PANDA), data modalities (e.g., EEG/MEG, fNIRS and MRI), species (e.g., humans, monkey and cat) and research fields (e.g., social networks and transportation networks). In particular, GRETNA allows researchers to preprocess human R-fMRI data and construct intrinsic functional brain networks.

**Figure 1:**
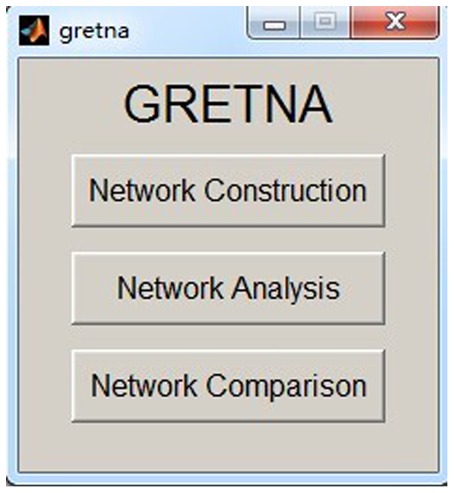
**The graphical user interface (GUI) of GRETNA.** The main window of GRETNA includes three panels: network construction, network analysis and network comparison.

**Figure 2:**
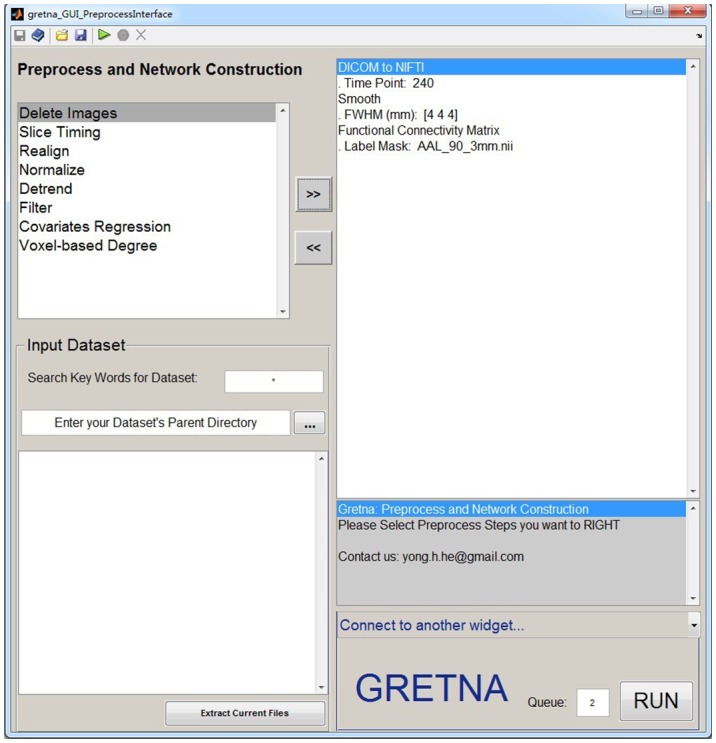
**The GUI panel of network construction.** In this panel, GRETNA allows researchers to perform all common preprocessing steps used by the R-fMRI community and construct large-scale functional brain networks using different region-based parcellations. Voxel-based degree centrality can also be computed here.

**Figure 3:**
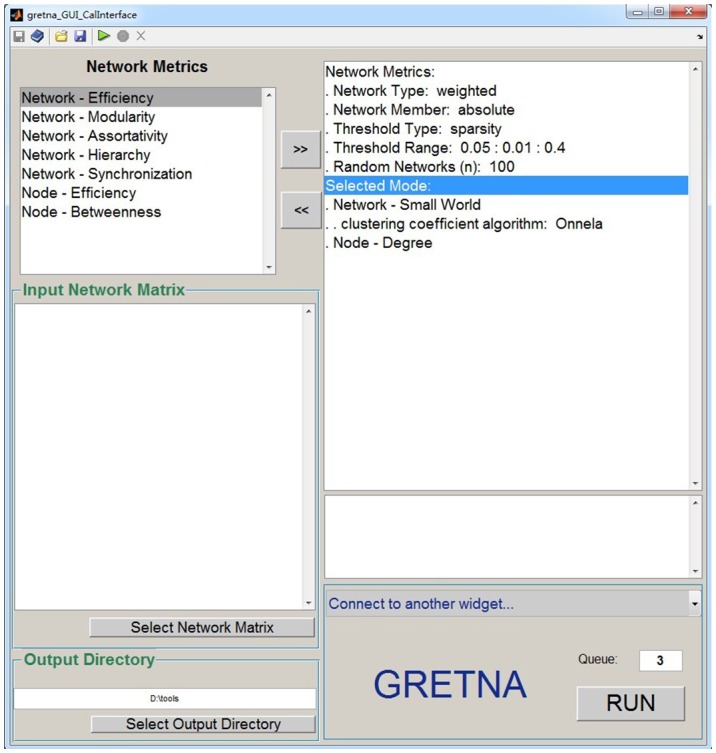
**The GUI panel of network analysis.** In this panel, GRETNA allows researchers to calculate many global and nodal graph-based metrics used in brain network studies. This panel provides flexible manipulations for researchers regarding the thresholding procedure, network type and network connectivity member. Notably, null random networks can be generated here to benchmark the results derived from brain networks.

**Figure 4:**
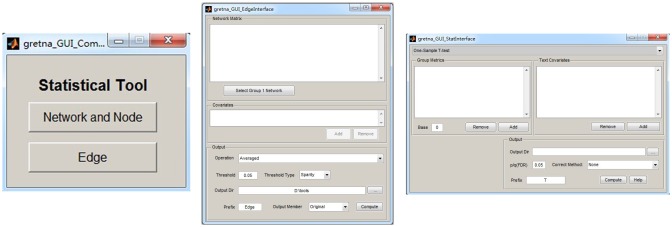
**The GUI panel of network comparison.** In this panel, GRETNA allows researchers to statistically infer effects of interest on network measures (global, nodal and connectional) using different parametric models and examine relationships between network measures and other variables (e.g., behavioral and clinical variables).

**Figure 5:**
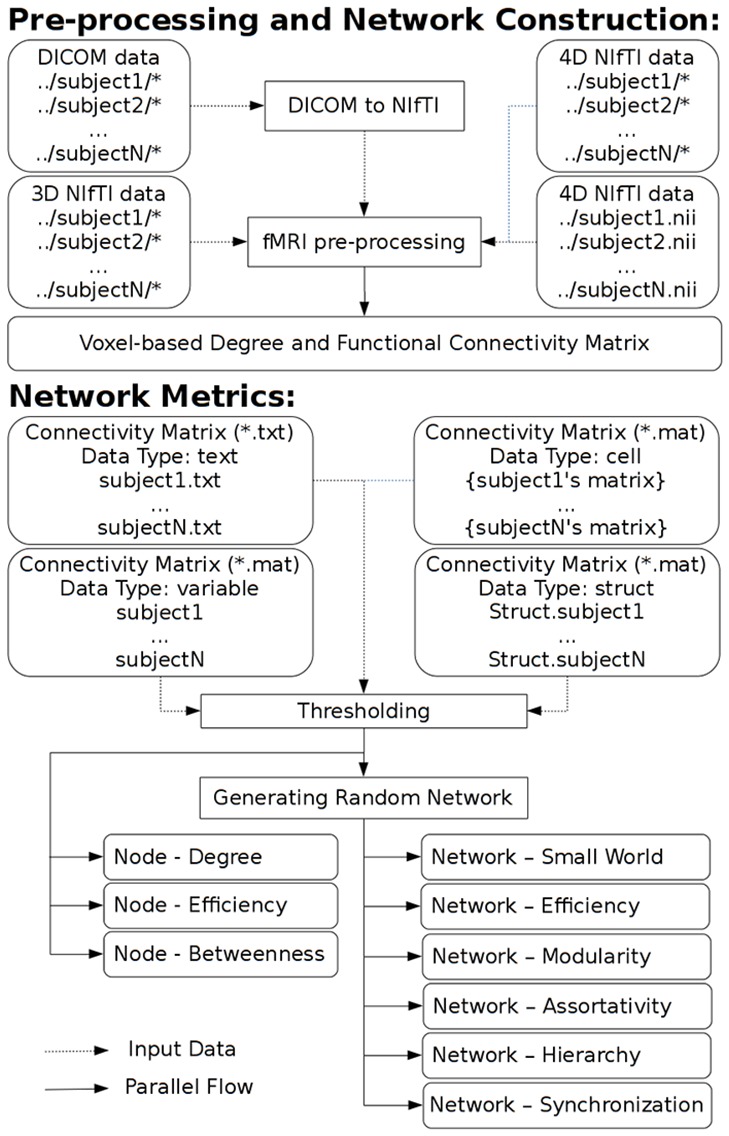
**A flowchart to explain brain network construction, topological characterization and parallel computing**.

GRETNA is divided into three sections: network construction, network analysis and network comparisons (Figure 1). In the network construction section, GRETNA allows researchers to: (i) perform R-fMRI data preprocessing, including volume removal, slice timing, realignment, spatial normalization, spatial smoothing, detrend, temporal filtering and removal of confounding variables by regression; (ii) compute voxel-based degree centrality (i.e., functional connectivity density); and (iii) construct region-based connectivity matrices (Figure 2). In this section, GRETNA accepts two types of data: DICOM data or Neuroimaging Informatics Technology Initiative (NIfTI) images (3D/4D). In the network analysis section, GRETNA allows researchers to: (i) convert individual connectivity matrices into a series of sparse networks according to the pre-assigned parameters of the network type (binary or weighted), network connectivity member (absolute, positive or negative), threshold type (connectivity strength or sparsity) and threshold range; (ii) generate benchmark random networks that match real brain networks in the number of nodes and edges and degree distribution; and (iii) calculate graph-based global and nodal network metrics (Figure 3). In this section, GRETNA accepts two types of data: text files (i.e., .txt) or Matlab data files (i.e., .mat). In the final network comparison section, GRETNA allows researchers to: (i) perform statistical inference on global, nodal and connectional network parameters; and (ii) estimate network-behavior relationships (Figure 4). It is worth highlighting that GRETNA executes parallel computing throughout R-fMRI data pre-processing, network construction and network parameter calculation by allotting processing tasks to different computational cores. This was done by calling the PSOM toolbox (Bellec et al., 2012) in a single PC. Of note, the parallel computing can work not only for multiple subjects, but also for a single subject when computing multiple network metrics. Figure 5 presents the flowchart of brain network construction and topological characterization and explains how parallel computing works. Below we describe these procedures in detail.

### Network Construction

In this section, GRETNA allows researchers to perform several preprocessing steps of R-fMRI data, that are commonly used in the community, and then construct large-scale brain networks by calculating the pairwise functional connectivity among a set of ROI according to a brain parcellation scheme. Notably, researchers can arbitrarily designate the order of preprocessing steps.

#### Data Format Conversion

Before formal data preprocessing, the DICOM data, a format output from most MRI scanners, is typically transformed into other formats, e.g., NIfTI format. Compared with the previous analyze file format, the NIfTI format contains new and important features, such as affine coordinate definitions that relate a voxel index to a spatial location, indicators of the spatial normalization type and records of the spatio-temporal slice ordering. This conversion is achieved in GRETNA by calling dcm2nii in the MRIcroN software.^2^

#### Removal of Volumes

The first several volumes of individual functional images are often discarded for magnetization equilibrium. GRETNA allows researchers to delete the first several volumes by specifying either the number of volumes to be deleted or the number of volumes to be retained. The latter is useful for across-datasets or across-center studies in which numbers of image volumes are usually different.

#### Slice Timing Correction

Currently, R-fMRI datasets are usually acquired using repeated 2D imaging methods, which leads to temporal offsets between slices. The slice-timing effects have been demonstrated to have prominent effects on study results and can be successfully compensated by the slice timing correction step (i.e., temporal data interpolation; Sladky et al., 2011). This is performed in GRETNA by calling the corresponding SPM8 functions. Of note, for a longer repeat time (e.g., > 3 s), within which a whole brain volume is acquired, it is advised to omit the slice time correction step because interpolation in this case becomes less accurate.

#### Realignment

During an MR scan, participants inevitably undergo various degrees of head movements even when foam pads are used. The movements break the spatial correspondence of the brain across volumes. This step realigns individual images so that each part of the brain in all volumes is in the same position. This is performed in GRETNA by calling relevant SPM8 functions.

#### Spatial Normalization

For group average and group comparison purposes, individual data are usually transformed into a standardized space to account for the variability in brain size, shape and anatomy. This can be accomplished in GRETNA by two methods based on the SPM8 functions: (i) directly warping individual functional images to standard MNI space by estimating their transformation to the echo-planar imaging (EPI) template (Ashburner and Friston, 1999); and (ii) warping individual functional images to standard MNI space by applying the transformation matrix that can be derived from registering the T1 image (co-registered with functional images) into the MNI template (Ashburner and Friston, 2005). The latter method tends to improve the accuracy of spatial normalization when the distortions of functional data are negligible, which is important to ensure effective cross-modality co-registration.

#### Spatial Smoothing

Smoothing, a common preprocessing step after spatial normalization, is used to improve the signal to noise ratio and attenuate anatomical variances due to inaccurate inter-subject registration. GRETNA performs spatial smoothing using a Gaussian filter with a shape that can be determined by a 3-value vector of full width at half maximum (FWHM) as implemented in SPM8.

#### Detrend

FMRI datasets may suffer from a systematic increase or decrease in the signal with time presumably due to long-term physiological shifts or instrumental instability (Lowe and Russell, 1999). GRETNA provides an option to reduce the effects of linear and non-linear drift or trend in the signal on the basis of relevant SPM8 functions. It should be noted that this step is still controversial (Smith et al., 1999) and researchers should interpret their results with caution if detrend is implemented.

#### Temporal Filtering

Previous studies have shown that spontaneous brain activity is predominantly subtended by the low-frequency components (0.01–0.1 Hz) of R-fMRI signals (Biswal et al., 1995; Lowe et al., 1998; Kiviniemi et al., 2000). Thus, R-fMRI data are typically band-pass filtered to reduce the effects of low frequency drift and high-frequency physiological noises. Notably, even in the typically used low-frequency intervals, accumulating evidence suggests that functional brain architectures are distinct across different frequency bands (Achard et al., 2006; Salvador et al., 2008; Zuo et al., 2010; Liao et al., 2013) and show frequency-specific alterations in neurological and psychiatric disorders, such as Alzheimer’s disease and mild cognitive impairment (Han et al., 2011; Wang et al., 2013; Liu et al., 2014). Moreover, recent studies highlight the physiological significance of high frequency fluctuations (Boubela et al., 2013; Liao et al., 2013). In GRETNA, we provide an option for researchers to easily choose the frequency ranges that the data will be filtered with an ideal box filter function. This is done by converting a time series into frequency domain using a Fast Fourier Transform (FFT), retaining amplitude spectrum for frequency components of interest and setting amplitude spectrum to 0 for other frequency components, and converting the new amplitude spectrum into time domain by an inverse FFT transform.

#### Removal of Confounding Variables

For R-fMRI datasets, several nuisance signals are typically removed from each voxel’s time series to reduce the effects of non-neuronal fluctuations, including head motion profiles, the cerebrospinal fluid (CSF) signal, the white matter (WM) signals and/or the global signal (Greicius et al., 2003; Fox et al., 2005). In GRETNA, researchers can assign any combination of these variables to be variables of no interest, which will be regressed out. By default, the global signal, CSF signal and WM signal are calculated within the BrainMask_05_61_73_61.img, the CsfMask_07_61_73_61.img and the WhiteMask_09_61_73_61.img, respectively. The three images are from the REST toolbox (Song et al., 2011) and separately correspond to brain masks of the whole brain, cerebral spinal fluid and WM in the standard MNI space. In addition, the first-order derivative of head motion profiles can also be removed.

#### Voxel-Based Degree

Degree is a measure that quantifies the importance/centrality of a node through the number and/or strength of connections to all other nodes in a network. Degree centrality has been widely used in brain network studies because it tends to have higher test-retest (TRT) reliability than other nodal centrality metrics (Wang et al., 2011; Cao et al., 2014), and it is well in line with physiological measures, such as the rates of cerebral blood flow and glucose metabolism (Liang et al., 2013; Tomasi et al., 2013). Three parameters are needed for voxel-based degree analysis based on R-fMRI data: (i) a brain mask to indicate the coverage of brain regions; (ii) a correlation threshold to exclude low-level correlations (e.g., 0.2); and (iii) a distance threshold to determine short/long connections. Using GRETNA, we can obtain a total of 18 voxel-based degree maps for each participant that vary across connectivity distance (i.e., short-, long- or full-range), sign (i.e., positive, negative or absolute) and type (i.e., binary or weighted). Researchers can choose these degree maps according to their research objectives.

#### Functional Connectivity Matrix

This option is used to construct individual interregional functional connectivity matrices in two major steps: regional parcellation (i.e., network node definition) and functional connectivity estimation (i.e., network edge definition). GRETNA provides options for several different parcellation schemes, including the structurally defined Anatomical Automatic Labeling atlas (AAL-90; Tzourio-Mazoyer et al., 2002) and Harvard-Oxford atlas (HOA-112; Kennedy et al., 1998; Makris et al., 1999) and the functionally defined Dos-160 (Dosenbach et al., 2006, 2010), Crad-200 (Craddock et al., 2012), Power-264 (Power et al., 2011) and Fair-34 (Fair et al., 2009). Additionally, GRETNA also contains functions that can be used to parcel the brain into an arbitrary number of ROIs with same or different sizes (Zalesky et al., 2010b). These parcellation approaches provide flexible choices to determine network nodes for specific research objectives and allow researchers to test the robustness of their findings across different regional parcellations (Wang et al., 2013). Once a parcellation scheme is chosen, a mean time series will be extracted from each parcellation unit, and pairwise functional connectivity is then estimated among the time series by calculating linear Pearson correlation coefficients. This will generate an N X N correlation matrix, with N being the number of regions included in the selected brain parcellation for each participant. Of note, this section also allows researchers to construct dynamic correlation matrix based on a sliding time-window approach.

### Network Analysis

In this section, GRETNA can calculate various topological properties of a network or graph from both global and nodal aspects, which can be compared with counterparts of random networks to determine the non-randomness.

#### Thresholding

Prior to topological characterization, a thresholding procedure is typically applied to exclude the confounding effects of spurious relationships in interregional connectivity matrices. Two thresholding strategies are provided in GRETNA: the absolute connectivity strength threshold and relative sparsity threshold (He et al., 2009). Specifically, for the connectivity strength threshold, researchers can define a threshold value such that network connections with weights greater than the given threshold are retained and others are ignored (i.e., set to 0s). This connectivity strength threshold method allows for the examination of the absolute network organization. Note that the same connectivity strength threshold usually leads to a different number of edges in the resultant networks, which could confound between-group comparisons in network topology (van Wijk et al., 2010). To address this problem, GRETNA provides an alternative threshold method—sparsity or density threshold. Sparsity is defined as the ratio of the number of actual edges divided by the maximum possible number of edges in a network. For networks with the same number of nodes, the sparsity threshold ensures the same number of edges for each network by applying a subject-specific connectivity strength threshold and therefore allowing an examination of relative network organization (He et al., 2009). These two thresholding strategies are complementary and together provide a comprehensive method to test the network organization. Finally, given the absence of definitive way in selecting a single threshold, researchers can input a range of continuous threshold values to study network properties in GRETNA.

#### Network Type

Networks can be binarized or weighted depending on whether the connectivity strength is taken into account. Previous brain network studies have mainly focused on binary networks due to the reduction in computational complexity and clearness of network metric definitions. Notably, binary networks neglect the strength of connections above the threshold, and therefore fail to identify subtle network organizations (Cole et al., 2010). In GRETNA, all network analyses can be conducted for both binary and weighted networks. Briefly, a connectivity matrix C_*ij*_ = [c_*ij*_]can be converted into either a binary network

(1) $$A_{ij}=[a_{ij}]=\{\begin{array}{l} 1,if|c_{ij}|>r_{thr};\\ 0,others \end{array}$$

or a weighted network

(2) $$W_{ij}=[w_{ij}]=\{\begin{array}{l} |c_{ij}|,if|c_{ij}|>r_{thr};\\ 0,others \end{array}$$

where r_*thr*_ is a connectivity strength threshold that is the same across all subjects for the connectivity strength thresholding procedure or a subject-specific connectivity strength threshold determined by the sparsity thresholding procedure. It should be emphasized that for weighted network analysis, the connectivity strength must reflect similarity (e.g., correlation coefficient) because the reciprocal of connectivity strength is used to calculate inter-nodal path length.

#### Network Connectivity Member

Previous R-fMRI studies have found that certain functional systems are anti-correlated (i.e., have a negative correlation) in their spontaneous brain activity (Greicius et al., 2003; Fox et al., 2005). However, negative correlations may also be introduced by global signal removal, a preprocessing step that is currently controversial (Fox et al., 2009; Murphy et al., 2009; Weissenbacher et al., 2009; Schölvinck et al., 2010). For network topology, negative correlations may have detrimental effects on TRT reliability (Wang et al., 2011) and exhibit organizations different from positive correlations (Schwarz and McGonigle, 2011). Accordingly, GRETNA provides options for researchers to determine the network connectivity members, based on which subsequent graph analyses are implemented: positive network (composed of only positive correlations), negative network (composed of only absolute negative correlations) or full network (composed of both positive correlations and the absolute values of the negative correlations).

#### Random Networks

Brain networks are typically compared with random networks to test whether they are configured with significantly non-random topology. In GRETNA, the random networks are generated by a Markov-chain algorithm (Maslov and Sneppen, 2002; Sporns and Zwi, 2004), which preserves the same number of nodes and edges and the same degree distribution as the real brain networks. Specifically, for a binary network, two edges (*i*_1_,*j*_1_) and (*i*_2_,*j*_2_), are first selected at random that is node *i*_1_ is connected to node *j*_1_ and node *i*_2_ is connected to node *j*_2_. If there are no edges between node *i*_1_ and node *j*_2_ and between node *i*_2_ and node *j*_1_, we then add two new edges, (*i*_1_,*j*_2_) and (*i*_2_,*j*_1_), to replace the original two edges, (*i*_1_,*j*_1_) and (*i*_2_,*j*_2_). This procedure is repeated 2 X the number of edges in the reference brain network to assure the randomized organization. For a weighted network the randomization is performed in a similar manner but in this case the weights are bound to the edges. It should be noted that how to generate random networks is an ongoing topic for brain network studies Zalesky et al. (2012); Hosseini and Kesler (2013). Therefore we also provide codes to generate random networks based on a time series randomization and correlation matrix randomization as introduced in Zalesky et al. (2012). Further studies are needed to produce null models that are more biologically meaningful as benchmarks for real brain networks.

#### Network Metrics

GRETNA can calculate several widely used network metrics in brain network studies for both binary and weighted networks. Generally, these measures can be categorized into global and nodal metrics. Global metrics include small-world parameters clustering coefficient and characteristic path length (Watts and Strogatz, 1998; Onnela et al., 2005), local efficiency and global efficiency (Latora and Marchiori, 2001, 2003), modularity (Newman, 2006), assortativity (Newman, 2002; Leung and Chau, 2007), synchronization (Barahona and Pecora, 2002; Motter et al., 2005) and hierarchy (Ravasz and Barabási, 2003). Nodal metrics include nodal degree, nodal efficiency (Achard and Bullmore, 2007) and nodal betweenness centrality (Freeman, 1977). Of note, during the calculation of the characteristic path length, local efficiency, global efficiency, nodal efficiency and betweenness, GRETNA computes the pairwise shortest path length matrix by calling functions from the MatlabBGL toolbox (version 4.0)^3^ (Floyd-Warshall algorithm for networks with density larger than 10% and Johnson’s algorithm otherwise). Additionally, GRETNA calculates the characteristic path length as the “harmonic mean” distance between all possible node pairs (Newman, 2003) to address the disconnected nodes. For the formula, usage and interpretation of these measures, see Rubinov and Sporns (2010) and Wang et al. (2011). Finally, GRETNA can also calculate the area under the curve (AUC) for each network measure to provide a scalar that does not depend on specific threshold selection (Wang et al., 2009; Zhang et al., 2011). It should be noted that this module can perform topological analysis of brain networks, independent of imaging modality and species. For example, the structural brain connectivity matrices in humans or macaques that are obtained from the PANDA software (Cui et al., 2013) or the CoCoMac database^4^ can be entered into the module for graph analysis.

### Network Comparison

In this section, GRETNA allows researchers to perform statistical testing on global, nodal and connectional network measures. For global and nodal network measures, GRETNA provides several popular parametric models, including one-sample *t*-test, two-sample *t-test*, paired *t-test*, one-way analysis of variance (ANOVA) and repeated measures ANOVA. GRETNA also provides multiple comparison correction approaches, including the false discovery rate (FDR) and Bonferroni procedures. With respect to inter-nodal connection comparisons, one-sample *t-test* and two-sample *t-test* are provided, followed by multiple comparison correction procedures with FDR, Bonferroni or network-based statistic methods (Zalesky et al., 2010a). Finally, the statistical analysis of network-behavior correlation can be implemented in this section. In addition, covariates of no interests (e.g., age, gender and clinical variables) can be added into all of these statistical models.

### Example R-fMRI Data to Illustrate the Usage of GRETNA

#### Participants and Data Acquisition

A publicly available TRT reliability dataset^5^ was employed to exemplify the usage of GRETNA. This dataset contains 57 healthy young volunteers in total (male/female: 30/27; age: 19–30 years) who completed two MRI scan sessions within an interval of approximately 6 weeks (40.94 ± 4.51 days). All participants were right-handed and had no history of neurological and psychiatric disorders. For the R-fMRI scans, participants were instructed to rest and relax with their eyes closed without falling asleep. Each R-fMRI scan includes 200 contiguous EPI functional volumes: time *repetition*_(TR)_ = 2000 ms; time *echo*_(TE)_ = 30 ms; flip *angle*_(FA)_ = 90°; number of slices = 33; slice thickness = 3.5 mm; slice gap = 0.7 mm; matrix = 64 × 64; and field of *view*_(FOV)_ = 200 × 200 mm^2^. Additionally, a high-resolution T1-weighted magnetization prepared gradient echo (MPRAGE) sequence was also obtained: TR = 2530 ms; TE = 3.39 ms; inversion time = 1100 ms; FA = 7°; number of slices = 144; slice thickness = 1.3 mm; slice gap = 0.65 mm; matrix = 256 × 192; and FOV = 256 × 256 mm^2^. Only the first session was used in the current study to explain the use of GRETNA. Of note, four participants were excluded due to excessive head motion or image quality (Dai et al., 2014).

#### Data Preprocessing

Data preprocessing included removal of the first 10 volumes, slice timing correction, head movement correction, spatial normalization (T1 segmentation), removal of linear trend, temporal band-pass filtering (0.01–0.1 Hz) and nuisance signal regression (24-parameter head motion profiles, global signal, CSF signal and WM signal).

#### Network Construction and Analysis

We first obtained 6 voxel-wise functional connectivity strength maps (i.e., voxel-based degree centrality maps) for each participant, which were combinations between network type (binary or weighted) and network connectivity member (positive, negative or absolute of both). We then constructed 6 inter-regional functional connectivity matrices for each participant according to the 6 different regional parcellation approaches provided in GRETNA (i.e., Power-264, Crad-200, Dos-160, Fair-34, AAL-90 and HOA-112). The order, location and name of each node under these parcellation atlases are provided in the toolbox (…\GRETNA\Templates)., These connectivity matrices were subsequently averaged across participants to derive 6 group-level mean connectivity matrices. These group-level matrices were further converted into a set of binary and weighted networks via connectivity strength (i.e., correlation) and sparsity thresholding procedures (both ranged from 0–1 with an interval of 0.04). Finally, we calculated various global (clustering coefficient, characteristic shortest path length, local efficiency, global efficiency, assortativity, hierarchy, synchronization and modularity) and nodal (nodal degree, nodal efficiency and nodal betweenness centrality) topological properties of these brain networks.

All imaging preprocessing, network construction and analyses were performed in the GRETNA toolbox. The results of the network analysis were visualized using the BrainNet Viewer toolbox (Xia et al., 2013).

## Results

### Voxel-Based Functional Connectivity Strength Maps

**Figure 6:**
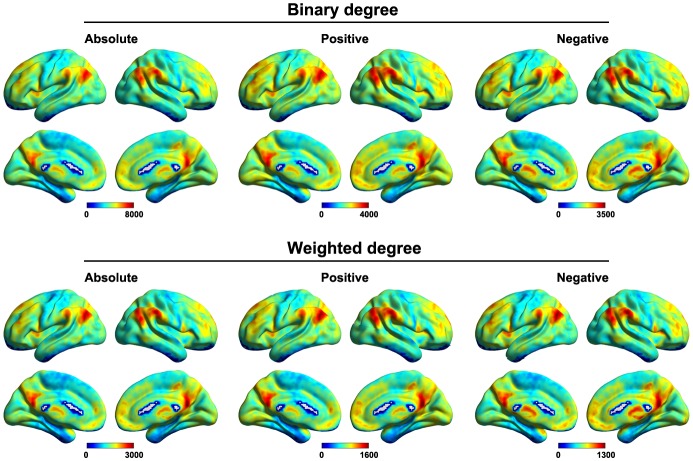
**Mean voxel-based functional connectivity strength.** Higher connectivity strength was observed primarily in the posterior cingulate gyrus, precuneus, medial prefrontal cortex, dorsolateral prefrontal cortex and subcortical structures. This pattern was generally robust across of network type (binary or weighted) and network connectivity member (absolute, positive or negative).

Figure 6 shows the mean voxel-based functional connectivity strength maps for all of the participants. We found that the functional connectivity strength was distributed heterogeneously over the brain with the most highly connected regions in the posterior cingulate gyrus, precuneus, medial prefrontal cortex, dorsolateral prefrontal cortex and subcortical structures (e.g., hippocampus, thalamus and amygdala). This pattern was generally robust across of network type (binary or weighted) and network connectivity member (absolute, positive or negative).

### Region-based Brain Networks: Global Metrics

**Figure 7:**
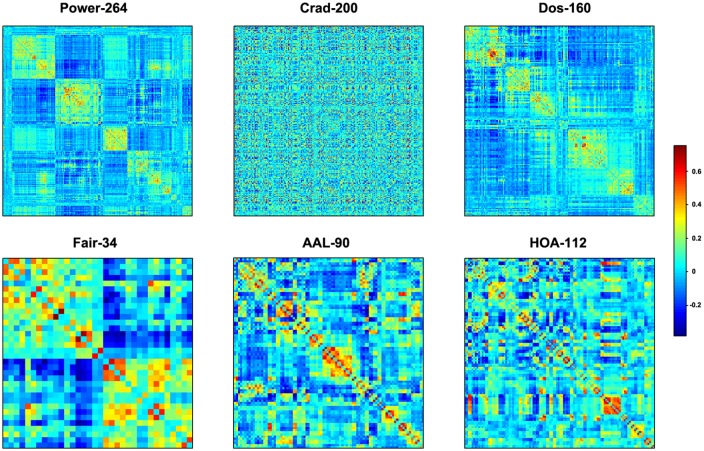
**Mean inter-regional correlation matrices.** Individual R-fMRI functional connectivity matrices were first transformed into z-score matrices (Fisher’s r-to-z transformation), then averaged across all participants, and finally inversely transformed into r-value matrices (Fisher’s r-to-z inverse transformation). Six different regional parcellation approaches were used, including four functionally defined parcellations (Power-264, Crad-200, Dos-160 and Fair-34) and two structurally defined parcellations (AAL-90 and HOA-112).

**Figure 8:**
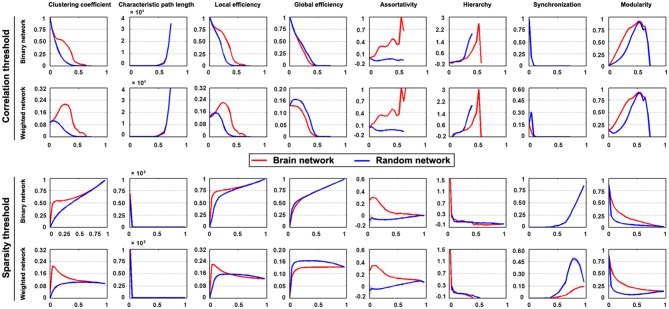
**Global organization of group-based functional brain network.** Significantly different organization was observed for R-fMRI brain networks from matched random networks, as characterized by a higher clustering coefficient, characteristic path length, local efficiency, assortativity and modularity but lower global efficiency. These findings were generally robust against the choices of network type and thresholding procedure.

The mean interregional functional connectivity matrices derived under each regional parcellation scheme are shown in Figure 7. Given the fact that: (i) the R-fMRI data were mainly used to illustrate the usage of GRETNA; (ii) the analyzed network properties have been frequently studied under both healthy and pathological conditions (For relevant reviews, see Bullmore and Sporns, 2009; He and Evans, 2010; Stam, 2014); and (iii) our findings were largely comparable with previous studies and were qualitatively independent of the brain parcellation schemes used in the current study; we thus only took Power-264 as an example to present our findings since this parcellation provided the highest spatial resolution among the 6 atlases used. Figure 8 presents all global metrics (clustering coefficient, characteristic path length, local efficiency, global efficiency, assortativity, hierarchy, synchronization and modularity) for both the group-based brain network and the 100 matched random networks as a function of sparsity and correlation thresholds. The functional brain network exhibited different organization from random networks, as characterized by a higher clustering coefficient, characteristic path length, local efficiency, assortativity and modularity but lower global efficiency. Most of these findings were robust against the selection of network types and threshold procedures. Additionally, several network measures varied depending on the choices of network type or thresholding procedure. For example, only weighted network analysis revealed lower synchronization for the brain network than the random networks; a hierarchical structure was evident in the brain network only when the correlation-based thresholding method was used.

### Region-Based Brain Networks: Nodal Metrics

**Figure 9:**
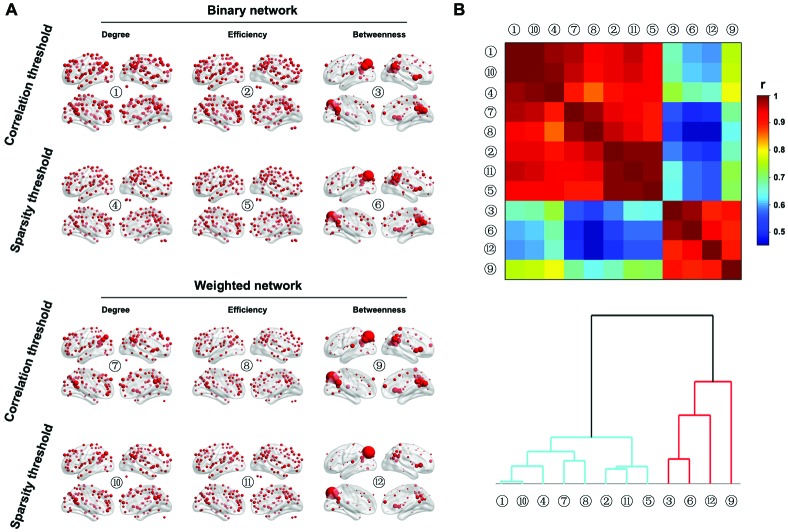
**Nodal characteristics of group-based functional brain network. (A)** Nodal degree, efficiency and betweenness were computed for both binary and weighted R-fMRI brain networks under both the correlation and sparsity thresholding procedures (only nodes with centralities larger than the mean of the whole brain network are shown). **(B)** Although significant correlations were observed in the spatial distributions among the three nodal centrality measures regardless of network type and threshold procedure, nodal betweenness revealed unique patterns compared to nodal degree and efficiency, which was demonstrated by the hierarchical clustering analysis.

Figure 9A shows the spatial distributions of three nodal centralities (degree, efficiency and betweenness) for both binary and weighted brain networks under both correlation and sparsity thresholding procedures (the AUCs were used here). The spatial distributions of nodal degree and efficiency were highly similar regardless of network type and thresholding procedure. Specifically, the posterior parietal, medial and lateral prefrontal and lateral temporal cortices as well as several subcortical structures exhibited the highest values. However, nodal betweenness exhibited obviously different patterns in that only the posterior parietal cortex showed extremely high betweenness in the brain, a consistent finding across different network types and thresholding procedures. Further clustering analysis of the spatial similarity (i.e., correlation) matrix of nodal centrality distributions validated this observation that betweenness centrality was separated from nodal degree and efficiency, which were clustered together (Figure 9B).

## Discussion

We developed a toolbox, GRETNA, to automatically analyze topological properties of brain networks that are not constrained by data modality and species. Specifically, GRETNA can perform R-fMRI data preprocessing, construct brain functional networks and calculate most commonly used global and nodal topological attributes with parallel computing ability. Moreover, GRETNA is flexible in dealing with several important methodological issues, such as network node definition, network types, thresholding procedure and treatment of negative correlations, all of which are great concerns in brain network studies. Finally, we utilized a publicly released R-fMRI dataset to demonstrate the capabilities of GRETNA.

Graph-based topological analysis of human brain networks is one of the most active domains in modern brain science. With the explosion of brain network studies, a growing number of toolboxes are being developed to facilitate the progress from brain network construction to topological characterization and result visualization (Table 1). For example, the PANDA toolbox has been developed to construct large-scale structural brain networks based on diffusion MRI data (Cui et al., 2013); the BCT toolbox allows topological analysis of networks based on Matlab codes (Rubinov and Sporns, 2010); and the BrainNet Viewer can visualize brain networks (Xia et al., 2013). For R-fMRI, toolboxes also exist with functionality in data preprocessing, network construction or descriptions, such as the REST (Song et al., 2011), CONN (Whitfield-Gabrieli and Nieto-Castanon, 2012) and GAT (Hosseini et al., 2012). Of note, the CONN toolbox can also calculate some topological attributes of networks. However, it is important to note that the majority of these toolboxes either can only address a single module of brain network construction (e.g., PANDA) or network metric calculation (e.g., BCT), or lack the ability to support parallel computing, therefore inconvenient for conducting a complete, efficient brain network study. In contrast, GRETNA combines parallel computing with a whole pipeline of R-fMRI data pre-processing, network construction and network topological characterization, which could significantly accelerate the research process during connectome studies. Specifically, compared with the recent developed GraphVar (Kruschwitz et al., 2015), GRETNA has distinct features in parallel computing, capability to preprocess R-fMRI data. In addition, connectome-based studies are of high complexity during their implementations as reflected by liberal choices in the analytical strategies, such as brain node and edge definition, thresholding procedure, network type and others. Due to the current lack of a gold standard in the determination of these options, GRETNA thus provides many options to address increasingly concerning issues in brain network analysis, such as the brain parcellation scheme, binary or weighted network type, thresholding procedure and treatment of negative correlations. This enables researchers to flexibly determine their analytical strategies and thus allow testing the robustness of their findings against different choices. Finally, the outputs from GRETNA are easily compatible with our previous connectome visualization tool, BrainNet Viewer (Xia et al., 2013).

Using a publicly released TRT dataset, we found that the most highly connected regions in the brain were predominantly in the posterior cingulate gyrus, precuneus, medial prefrontal cortex, dorsolateral prefrontal cortex and several subcortical structures. This finding is generally robust against the spatial resolutions (voxel- or region-level) and centrality measures (degree, efficiency or betweenness) used, particularly for the posterior parietal regions. These identified hubs are comparable with previous structural and functional brain network studies (Hagmann et al., 2008; Buckner et al., 2009; Gong et al., 2009; Tomasi and Volkow, 2010; Liang et al., 2013). Moreover, the hub topography was independent of several factors of network type, network connectivity member and thresholding procedure, indicating that hubs are a stable, intrinsic property of brain network architecture. Of note, despite high spatial correlations, nodal betweenness behaved differently from nodal degree and efficiency in capturing hub topography, presumably due to their differences in depending on only one graph property (i.e., first-order; degree and efficiency) or on more than one property or ratios of one property (i.e., second-order; betweenness; Wang et al., 2011).

At the global level, the human brain networks exhibit different organization from matched random networks as characterized by a higher clustering coefficient, characteristic path length, local efficiency, assortativity and modularity and lower global efficiency, which is indicative of the efficient small-world, assortative and modular organizations of functional brain networks. This is consistent with numerous previous brain networks studies (Park et al., 2008; Bullmore and Sporns, 2009; He and Evans, 2010; Meunier et al., 2010; Braun et al., 2012; Liang et al., 2012). Additionally, these findings were robust against the factors of network connectivity member and thresholding procedure, suggesting that these organizational principles are stable configurations embedded in the functional brain networks. Regarding hierarchy, positive values were observed, which indicates a hierarchical structure of functional brain networks. In hierarchical networks, highly connected hubs tend to link nodes that have a limited chance to interconnect with each other, which favors top-down routing among network nodes on the one hand and minimize wiring costs on the other hand (Ravasz and Barabási, 2003). The hierarchical structure observed here is consistent with previous brain network studies (Bassett et al., 2008; Braun et al., 2012; Liang et al., 2012). Additionally, we also noted positive synchronization for functional brain networks, a feature that has been relatively less studied in human brain networks than other measures. Notably, the behaviors of hierarchy and synchronization seemed to depend on the analytical strategies: that more obvious deviations of functional brain networks from matched random networks appeared when the weighted network analysis was used for synchronization and the correlation-based thresholding procedure was used for hierarchy. Taken together, GRETNA revealed largely comparable findings with previous brain network studies, therefore demonstrating its effectiveness.

It should be noted that while graph-based brain network studies are burgeoning, they are still in their infancy. There are many methodological challenges that remain elucidative, such as head motion correction (Muschelli et al., 2014; Patel et al., 2014), null model construction (Zalesky et al., 2012; Hosseini and Kesler, 2013), thresholding method selection (Toppi et al., 2012) and connectivity type determination (Salvador et al., 2007; Liang et al., 2012). Moreover, there are certain topological attributes that are not included in the current GRETNA, such as rich-club architecture (van den Heuvel and Sporns, 2011) and motif (Milo et al., 2002). Future versions of GRETNA will expand the functionality of these aspects. GRETNA can be further improved by integrating independent component analysis to allow exploring functional brain network topology among different brain components or subsystems (Yu et al., 2011, 2013) and sophisticated methods to characterize temporal evolution of functional brain networks (Liao et al., 2014; Zalesky et al., 2014) or both (Yu et al., 2015) In addition, the current GRETNA can only handle undirected networks (binary and weighted). Recent methodological advances have allowed researchers to infer large-scale directed brain networks with R-fMRI data (Liao et al., 2011; Yan and He, 2011). Hence, an important future extension of GRETNA is to add functionality to address directed networks. Finally, although the current GUI version of GRETNA includes several statistical functions, they are all parametric. Given the lack of statistical theory regarding the distribution of graph metrics for human brain networks, future versions could contain nonparametric inference of brain network metrics (Bullmore and Sporns, 2009), such as the permutation test (Wang et al., 2013), Functional Data Analysis (Bassett et al., 2012) or re-sampling approach (Gong et al., 2012).

In conclusion, we developed a user-friendly and easily navigable toolbox, GRETNA, to assist in conducting topological analysis of structural and functional brain networks. This toolbox has a highly compatible GUI with the widely used SPM toolbox. We hope that the toolbox contributes to facilitating and standardizing brain connectomics studies based on graph theory.

## Author Contributions

Conceived and designed the study: JW, XW, AE and YH. Performed the study: JW, XW and YH. Analyzed the data: JW, XL and MX. Contributed reagents/materials/analysis tools: JW, XW, AE and YH. Wrote the paper: JW and YH.

## Conflict of Interest Statement

The authors declare that the research was conducted in the absence of any commercial or financial relationships that could be construed as a potential conflict of interest.

## Footnotes

^2^http://www.mccauslandcenter.sc.edu/mricro/mricron/

^3^https://www.cs.purdue.edu/homes/dgleich/packages/matlab_bgl/

^4^http://cocomac.g-node.org/

^5^http://fcon_1000.projects.nitrc.org/indi/CoRR/html/bnu_1.html
