# Corrigendum: GRETNA: a graph theoretical network analysis toolbox for imaging connectomics

**DOI:** 10.3389/fnhum.2015.00458

**Published:** 2015-08-19

**Authors:** Jinhui Wang, Xindi Wang, Mingrui Xia, Xuhong Liao, Alan Evans, Yong He

**Affiliations:** ^1^State Key Laboratory of Cognitive Neuroscience and Learning and IDG/McGovern Institute for Brain Research, Beijing Normal UniversityBeijing, China; ^2^Center for Cognition and Brain Disorders, Hangzhou Normal UniversityHangzhou, China; ^3^Zhejiang Key Laboratory for Research in Assessment of Cognitive ImpairmentsHangzhou, China; ^4^McConnell Brain Imaging Center, Montreal Neurological InstituteMontreal, QC, Canada

**Keywords:** network, graph theory, connectome, resting fMRI, small-world, hub

Here, we would like to correct the two points as follows.

1) The updated Table 1:

**Table 1:** **Summary of neuroscience connectomics tools**.

**Software**	**R-fMRI preprocessing**	**Network construction (static)**	**Network construction (dynamic)**	**Graph analysis**	**Statistics**	**Fle**	**GUI**	**Parallel computing**	**Vis**	**Website**
GRETNA	✓	✓	✓	✓	✓	✓	✓	✓	×	http//www.nitrc.org/projects/gretna/
BCT	×	×	×	✓	×	×	×	×	×	https://sites.google.com/site/bctnet/
GAT	×	✓	×	✓	✓	×	✓	×	✓	Not available
PANDA	×	✓	×	×	×	×	✓	✓	×	http//www.nitrc.org/projects/panda/
CONN	✓	✓	×	✓	✓	×	✓	×	✓	http//www.nitrc.org/projects/conn
eConnectome	×	✓	×	×	×	×	✓	×	✓	http://econnectome.umn.edu/
BrainNel Viewer	×	×	×	×	×	×	✓	×	✓	http://www.nitrc.org/projects/bnv/
GraphVar	×	✓	✓	✓	✓	✓	✓	✓	✓	http://www.nitrc.org/projects/graphvar/
Brainwaver	×	✓	×	✓	×	×	×	×	✓	http://cran.r-project.org/web/packages/brainwaver/

*Fie, flexibility; GUI, graphical user interface; Vis, visualization. Note: flexibility is determined according to whether a toolbox provides options regarding at least three of the following factors: network node, network connectivity, network connectivity member, network type and thresholding procedure*.

2) Discussion: “Specifically, compared with the recent developed GraphVar (Kruschwitz et al., 2015), GRETNA has distinct features in parallel computing, capability to preprocess R-fMRI data.”

We would like to further clarify the description as

“Specifically, compared with the recent developed GraphVar (Kruschwitz et al., 2015), GRETNA has distinct features in parallel computing. The GraphVar (beta v0.611) can assign several jobs to different CPUs by calling Matlab's parallel computing toolbox. The GRETNA can assign parallel tasks by calling the PSOM toolbox (Bellec et al., 2012), which helps GRETNA to record and manage the data generated during fMRI preprocessing or graph-based network analyses and to restart the pipeline from the failure steps.”

## Conflict of interest statement

The authors declare that the research was conducted in the absence of any commercial or financial relationships that could be construed as a potential conflict of interest.
